# The role of an anti-inflammatory molecule AIM/CD5L in gut ischemia/reperfusion injury of male mice

**DOI:** 10.1186/s10020-025-01385-1

**Published:** 2025-10-29

**Authors:** Russell Hollis, Gaifeng Ma, Alok Jha, Megan Tenet, Takayuki Kato, Monowar Aziz, Ping Wang

**Affiliations:** 1 Center for Immunology and Inflammation, The Feinstein Institutes for Medical Research, Manhasset, NY USA 350 Community Dr., 11030; 2 Department of Surgery, Zucker School of Medicine, Manhasset, NY USA; 3 Elmezzi Graduate School of Molecular Medicine, Manhasset, NY USA

**Keywords:** Gut ischemia, Reperfusion injury, Apoptosis inhibitor of macrophage, CD5L, Damage-Associated molecular pattern

## Abstract

**Introduction:**

Resolution of acute gut ischemia causes reperfusion injury, resulting in the release of damage-associated molecular patterns (DAMPs) and tissue injury. A key DAMP, extracellular cold-inducible RNA-binding protein (eCIRP), exacerbates inflammation in reperfusion injury, contributing to organ failure and death. Apoptosis inhibitor of macrophage (AIM or CD5L) is a glycoprotein secreted by macrophages which can influence the activity of immune cells. We seek to investigate AIM expression in ischemia/reperfusion (I/R) and elucidate its anti-inflammatory role in macrophages and intestinal epithelial cells.

**Methods:**

Male mice underwent occlusion of the superior mesenteric artery for 60 min, followed by reperfusion for 4 h before sample collection. AIM expression in blood and tissue was evaluated by qPCR, Western blot, and ELISA. Primary peritoneal macrophages from male mice, IEC-6 intestinal epithelial cells, and RAW 264.7 macrophages were stimulated with recombinant mouse (rm) CIRP (denoted eCIRP) and treated with rmAIM. Cytokine levels were assessed by ELISA and qPCR. Metabolic function was measured in macrophages using the Agilent Seahorse XF Pro analyzer. Interactions involving AIM, eCIRP, and eCIRP’s receptors, Toll-like receptor 4 (TLR4) and triggering receptor expressed on myeloid cells-1 (TREM-1), were elucidated by in silico approaches.

**Results:**

Pulmonary AIM mRNA expression decreased by 55.9% (*p* = 0.018), and protein levels decreased by 26.9% (*p* = 0.032) in gut I/R mice compared to sham mice. Plasma AIM concentration decreased by 22.0% (*p* = 0.0362) in gut I/R mice compared to sham. eCIRP treatment increased pro-inflammatory cytokine production by macrophages and intestinal epithelial cells. This increase was significantly attenuated by co-treatment with rmAIM. Macrophages also increased basal oxygen consumption rate by 66.7% and ATP production by 70.3% when treated with rmAIM compared to eCIRP stimulation alone (*p* < 0.0001). Computational modeling predicted strong interactions between AIM and eCIRP’s receptors, TLR4 and TREM-1, and showed that the presence of AIM altered eCIRP’s binding to these receptors.

**Conclusion:**

In male mice, gut I/R decreases AIM protein levels and mRNA expression in the lungs as well as AIM plasma concentration. AIM reduces eCIRP-induced pro-inflammatory cytokine production in macrophages, potentially by inhibiting eCIRP’s binding to TLR4 and TREM-1. These findings suggest AIM is a promising therapeutic candidate in males with gut I/R.

**Supplementary Information:**

The online version contains supplementary material available at 10.1186/s10020-025-01385-1.

## Introduction

Acute gut ischemia affects approximately 1 in every 1,000 hospitalized patients (Monita and Gonzalez 2023). The disease is highly morbid and often fatal, with most studies reporting a 60–80% mortality rate due to rapid disease progression from complete obstruction of the superior mesenteric artery (SMA) (Monita and Gonzalez 2023). Initial treatment involves resuscitation and anticoagulation, followed by mechanical removal of the occlusive agent via embolectomy or thrombectomy (Clair and Beach 2016). Once the obstruction is relieved, intestinal resection is performed if required (Clair and Beach 2016). However, after the obstruction is relieved, systemic circulation of byproducts from cell death and tissue ischemia, such as damage-associated molecular patterns (DAMPs), can result in reperfusion injury (Cowled and Fitridge 2011; Hollis et al. 2025). Reperfusion injury contributes to mortality from gut ischemia through end organ damage, including acute respiratory distress syndrome (ARDS) (Grootjans et al. 2016; Lv et al. 2024). One critical DAMP recently implicated as a driver of reperfusion injury is extracellular cold-inducible RNA-binding protein (eCIRP) (Aziz et al. 2025).

CIRP is an18 kDa RNA chaperone protein with important homeostatic functions (Aziz et al. 2019, 2025). During cellular stress or after cell death, CIRP is released into the extracellular space, acting as a ligand for toll-like receptor 4 (TLR4) and triggering receptor expressed on myeloid cells-1 (TREM-1) to promote cytokine expression, exacerbating inflammation (Aziz et al. 2025; Murao et al. 2021). eCIRP levels are elevated in the blood of critically ill patients with sepsis (Zhou et al. 2015) and in several animal models of disease, including hemorrhagic shock and ischemia/reperfusion (I/R) injury (Hollis et al. 2025; McGinn et al. 2018; Qiang et al. 2013). While inflammation may play a beneficial role in some capacity, an immune system dysregulated by excessive inflammation worsens outcomes (Singer et al. 2016). For example, eCIRP has been demonstrated in previous studies to exacerbate gut I/R injury with increased cytokine expression (Cen et al. 2017; Hollis et al. 2025; McGinn et al. 2018). Furthermore, CIRP^−/−^ mice have a reduced inflammatory response and improved outcomes in sepsis and gut I/R (Cen et al. 2017; Khan et al. 2017). Modulating inflammation through inhibiting eCIRP-receptor interactions is therefore a viable therapeutic strategy in gut I/R.

Apoptosis inhibitor of macrophage (AIM), also known as CD5L, is a glycoprotein secreted by macrophages that consists of three scavenger receptor cysteine-rich (SRCR) domains (Sanchez-Moral et al. 2021). AIM performs a variety of functions, including inhibition of apoptosis, promotion of autophagic mechanisms, uptake of oxidized lipoproteins, and clearance of necrotic cell debris through activation of scavenger receptors, such as CD36 and kidney injury molecule 1 (KIM-1) (Sanchez-Moral et al. 2021; Sanjurjo et al. 2015; Wang et al. 2021). A recent investigation of AIM in sepsis demonstrated increased inflammation in AIM^−/−^ mice and improved outcomes in mice treated with recombinant AIM (Oliveira et al. 2024). In addition, a negative association between AIM and DAMP concentrations has been observed, and the glycoprotein has been investigated as a scavenger of several DAMPs (Maehara et al. 2021; Oliveira et al. 2024). However, AIM’s status in gut I/R and its impact against eCIRP-mediated inflammation have not been explored.

We hypothesized that endogenous AIM protein and mRNA expression decrease during acute gut I/R in male mice and that AIM can inhibit eCIRP-induced inflammation in macrophages and intestinal epithelial cells, which both play critical roles in exacerbating gut I/R pathophysiology. To test this hypothesis, we first measured endogenous AIM protein and mRNA expression in a mouse model of acute gut I/R. Next, we determined the effects of recombinant mouse (rm) AIM on eCIRP-stimulated inflammation in macrophages and intestinal epithelial cells. Finally, we used computational modeling to investigate the potential mechanisms by which rmAIM could prevent eCIRP’s interaction with its target receptors, thereby inhibiting inflammation. These findings suggest AIM as a promising therapeutic candidate for males with gut I/R injury.

## Materials and methods

### Recombinant proteins and peptides

Recombinant murine (rm) CD5L was purchased from Creative Biomart (cat. Cd5l-659 M, Shirley, NY). rmCD5L has the same amino acid sequence as the endogenous protein but lacks the secretory signal, which is normally cleaved after secretion. The recombinant protein is synthesized in HEK293 cells to ensure glycosylation, which is consistent with the endogenous protein and reflected in the observed molecular weight of 50–60 kDa (predicted 38 kDa). Recombinant mouse CIRP (denoted as eCIRP) was synthesized and validated in our lab as previously described (Qiang et al. 2013).

### Experimental animals

Male C57BL/6 mice aged 8–13 weeks (wk) (Jackson Laboratory, Bar Harbor, ME and Charles River Laboratories, Wilmington MA) were housed in a standard environment and allowed at least 3 days to acclimate. All experiments were approved by the Institutional Animal Care and Use Committee (IACUC) at the Feinstein Institutes for Medical Research. Female mice were not used for this study to avoid confounding factors related to the differences between male and female response to I/R injury and inflammation (Homma et al. 2005; Hundscheid et al. 2020; Wu et al. 2021).

### Gut ischemia/reperfusion injury

Male C57BL/6 mice aged 8–13 weeks were subjected to either sham surgery or gut I/R as previously described (Hollis et al. 2025). In brief, mice were anesthetized with 2–4% inhalational isoflurane and monitored for respiration and absence of pedal reflex. A 1–2 cm laparotomy incision was made, and intestines were mobilized to expose the SMA, which was occluded with an atraumatic vascular clamp (Item No. 18055-04; Fine Science Tools, Foster City, CA) for 60 min (min). After 60 min, the clamp was removed, and the incision was closed. Mice were given 0.5 ml normal saline resuscitation and 0.05 mg/kg buprenorphine analgesia via subcutaneous injection. Mice were allowed to recover for 4 h (h) before specimen collection.

We established this gut I/R model in our lab’s previous work (Hollis et al. 2025; Kato et al. 2025; Kobritz et al. 2022; Royster et al. 2021), where we show appropriate injury after 60 min of ischemia and 4 h of reperfusion. In these previous publications, we validated the level of intestinal injury in this model with a frequently cited injury scoring system (Stallion et al. 2005). While ischemia times can vary across studies due to environmental and perioperative factors, our consistent prior data confirm that our model parameters reliably induce reperfusion injury without excessive tissue death.

### Cell culture and treatment

RAW 264.7 cells were cultured in Dulbecco’s modified eagle medium (DMEM; Thermo Fisher Scientific, Waltham, MA) supplemented with 10% fetal bovine serum (FBS, Thermo Fisher Scientific), 1% penicillin/streptomycin (PCN/Strep, Thermo Fisher Scientific), and 1% L-glutamine. These cells were used for Seahorse XF (Agilent Technologies, Santa Clara, CA) metabolic assays.

Primary macrophages were isolated from healthy adult, male C57BL/6 mice via peritoneal lavage using phosphate-buffered saline (PBS; Crystalgen, Commack, NY) supplemented with 2–5% FBS and 1% PCN/Strep. Peritoneal cavity macrophages were isolated via the adhesion method whereby cells were washed twice with warm PBS after a 2 h incubation period then cultured in Roswell Park Memorial Institute medium (RPMI; Thermo Fisher Scientific) supplemented with 10% FBS, 1% PCN/Strep, 2% L-glutamine, and 25 millimolar (mM) HEPES buffer (Thermo Fisher Scientific). Peritoneal cavity macrophages were used for protein expression experiments.

IEC-6 (ATCC, Manassas, Virginia) intestinal epithelial cells were cultured in DMEM supplemented with 10% FBS, 1% PCN/Strep, and 10 µg/ml human recombinant insulin (Thermo Fisher Scientific) and used for protein and mRNA expression experiments.

Cell cultures were incubated overnight in their respective media before treatment with eCIRP, rmAIM, or eCIRP with rmAIM in various doses for 4–24 h. Treatments were co-incubated at room temperature for 30 min before use in cell cultures. During the treatment period for respective cell cultures, the media was replaced with Opti-Mem reduced serum media (Thermo Fisher Scientific).

### ELISA

Plasma samples from male mice who underwent gut I/R and sham mice were isolated from blood via centrifugation and used for CD5L (AIM) ELISA. This ELISA was performed according to the manufacturer’s protocol using a 1:1000 sample dilution (Cat. KIT50020, Sino Biological, Beijing, China). For in vitro experiments, the supernatant of cultured peritoneal cavity macrophages (7.5 × 10^4^/well) and IEC-6 cells (3 × 10^5^/well) were collected from separate experiments after 4–24 h of treatment. ELISA for TNFα and IL-6 were performed for peritoneal cavity macrophages (Cat. 558534 and 555240, respectively, BD Biosciences, San Diego, CA) and IEC-6 cells (Cat. 558535 and 550319, respectively, BD Biosciences, San Diego, CA) according to the manufacturer’s protocol with overnight sample incubation at 4°C. TNFα and IL-6 were chosen to evaluate rmAIM’s effects on eCIRP as they are inflammatory cytokines known to increase after stimulation with eCIRP and are associated with pro-inflammatory (M1) macrophage polarization (Gurien et al. 2020; Shimizu et al. 2024).

### Real time quantitative polymerase chain reaction (RT qPCR)

Lung tissue samples were flash frozen in liquid nitrogen and stored at −80°C after gut I/R. For in vivo samples, mRNA was isolated with the TRIzol method (Thermo Fisher Scientific, Waltham, MA). For in vitro samples, RNA was isolated using the Cytiva RNAspin Mini Kit (Cat. 25050072, Marlborough, MA). Isolated mRNA was used for reverse transcription with a Veriti 96-well Thermal Cycler (Applied Biosystems, Foster City, CA) followed by RT qPCR with the StepOne Plus Real-Time PCR System (Applied Biosystems, Foster City, CA). Messenger RNA (mRNA) fold expression was calculated using the 2^−CtΔΔ^ method with β-actin as the reference gene. The following genes and primer sequences (forward and reverse, respectively) were used: β-actin: 5’-CGTGAAAAGATGACCCAGATCA-3’ and 5’-TGGTACGACCAGAGG CATACAG-3’; CD5L: 5’-GTTCAACTTGATGCTGGCCA-3’ and 5’-GCCATCATCACAC ACAGTCC-3’; TNFα: 5’-AGACCCTCACACTCAGATCATCTT C-3’ and 5’-TTGCTACGACGTGGG CTACA-3’; IL-6:5’-CCGGAGAGGAGACTTCACAG-3’ and 5’-GGAAATTGGGGTAG GAAGGA-3’; MIP-2: 5’-CGCCCAGACAGAAGTCATAG-3’ and 5’CCTTTCCAGGTCA GTTAGCC-3’; iNOS: 5’-GGAGAGAGATCCGGTTCACAGT-3’ and 5’-ACCTTCCGC ATTAGCACAGAA-3’.

### Western blot assay

Western blotting was performed on in vivo lung tissue, collected as previously described. Protein was isolated using complete radioimmunoprecipitation assay (RIPA) lysis buffer with 2 mM Na Orthovanadates, 0.2 mM PMSF, and complete mini protease inhibitor cocktail (Ref: 11836153001, Roche Diagnostics, Mannheim, Germany). 20 µg of protein was used per sample. The membranes were incubated overnight at 4 °C in primary antibody CD5L (D-11) at a 1:250 dilution (cat. sc-390486, Santa Cruz Biotechnolgy, Dallas, Texas) and GAPDH as the reference protein at a 1:5000 dilution (Cat. No.: 60004-1-1 g, Proteintech, Rosemont, Illinois). Membranes were incubated in secondary antibody for 1 h before washing and reading with an Odyssey imaging system (Li-Cor, Lincoln, Nebraska).

### Seahorse metabolic assay

Seahorse XF Pro analyzer and XF wave pro software (Agilent Technologies) were used to perform the Real-Time ATP rate and Mito Stress Test assays. RAW 264.7 cells were seeded at a density of 20,000 cells/well and incubated overnight. Cells were treated for 16 h using eCIRP at 1 µg/ml with or without CD5L 0.5,1, or 2 µg/ml 16 h before performing the assay. Media was exchanged with pre-warmed Seahorse XF RPMI (Cat. No.: 103576-100, Agilent Technologies) without Phenol Red supplemented with 2 mM glutamine, 10 mM glucose, and 1 mM pyruvate, and cells were placed in a non CO_2_ incubator for 60 min. Cells were then washed, and the XF Cell Mito Stress Test was performed with the following compound concentrations: Oligomycin: 1.5 µM, FCCP:1 µM, and Rotenone/Antimycin: 0.5 µM (Cat. No.:103015-100, Agilent Technologies).

### Computational modeling

The amino acid sequences of Mouse TLR4 (Q9QUK6), TREM-1 (Q9JKE2), CD5L (Q9QWK4) and CIRP (P60824) were retrieved from the Uniprot database. The models were generated using Iterative Threading ASSEmbly Refinement (I-TASSER) server (Yang et al. 2015) based on templates identified by threading approach to maximize percentage identity, sequence coverage, and confidence.

TLR4 is a transmembrane receptor that functions as a pattern recognition receptor for eCIRP, and TREM-1 is a cell surface receptor that also recognizes eCIRP. Both receptors promote downstream inflammatory cascades. The CIRP structure has different domains including RNA binding domain (aa 6–84), disordered region (aa 70–172), and polar residues (aa 143–172). AIM has asparagine residues N99 and N229, which are N-glycosylated.

Models were refined based on repetitive relaxations by short molecular dynamics simulations for mild (0.6 ps) and aggressive (0.8 ps) relaxations with 4 fs time step after structure perturbations. The model refinement enhanced certain parameters including Rama favored residues and decrease in poor rotamers. For TLR4-CD5L and TREM-1-CD5L or TREM-1-CD5L-CIRP and TLR4-CD5L-CIRP protein structure models, docking was performed using GRAMMX (Tovchigrechko and Vakser 2006) and ATTRACT tools (Schindler et al. 2015). The docking approach allowed simultaneous adjustment of side chain conformations and energy minimization in translational and rotational degrees of freedom of one protein with respect to another protein. The docking process included pre-calculation of potential energy on a grid, and then interactions were calculated by interpolation from the nearest grid points. Moreover, the docking process included several Monte Carlo simulations or energy minimization steps.

The analysis of TLR4-CD5L and TREM1-CD5L or TREM-1-CD5L-CIRP and TLR4-CD5L-CIRP complex interactions were calculated using PDBePISA tool (Krissinel and Henrick 2007). The surface area of interaction interfaces and thermodynamic parameters were calculated. The complex structures were visualized using PyMOL (Schrodinger, Inc., New York, NY) and Chimera (Sanner et al. 1996).

### Statistical analysis

Results were analyzed with GraphPad Prism (La Jolla, California). In vivo experiments were assessed using Student’s unpaired t-test. In vitro experiments were evaluated using one-way ANOVA, and each pair was analyzed using Tukey’s multiple comparisons test. A statistically significant difference was defined as *p* < 0.05.

## Results

### AIM decreases in the lungs and blood after gut ischemia/reperfusion in male mice

**Fig. 1:**
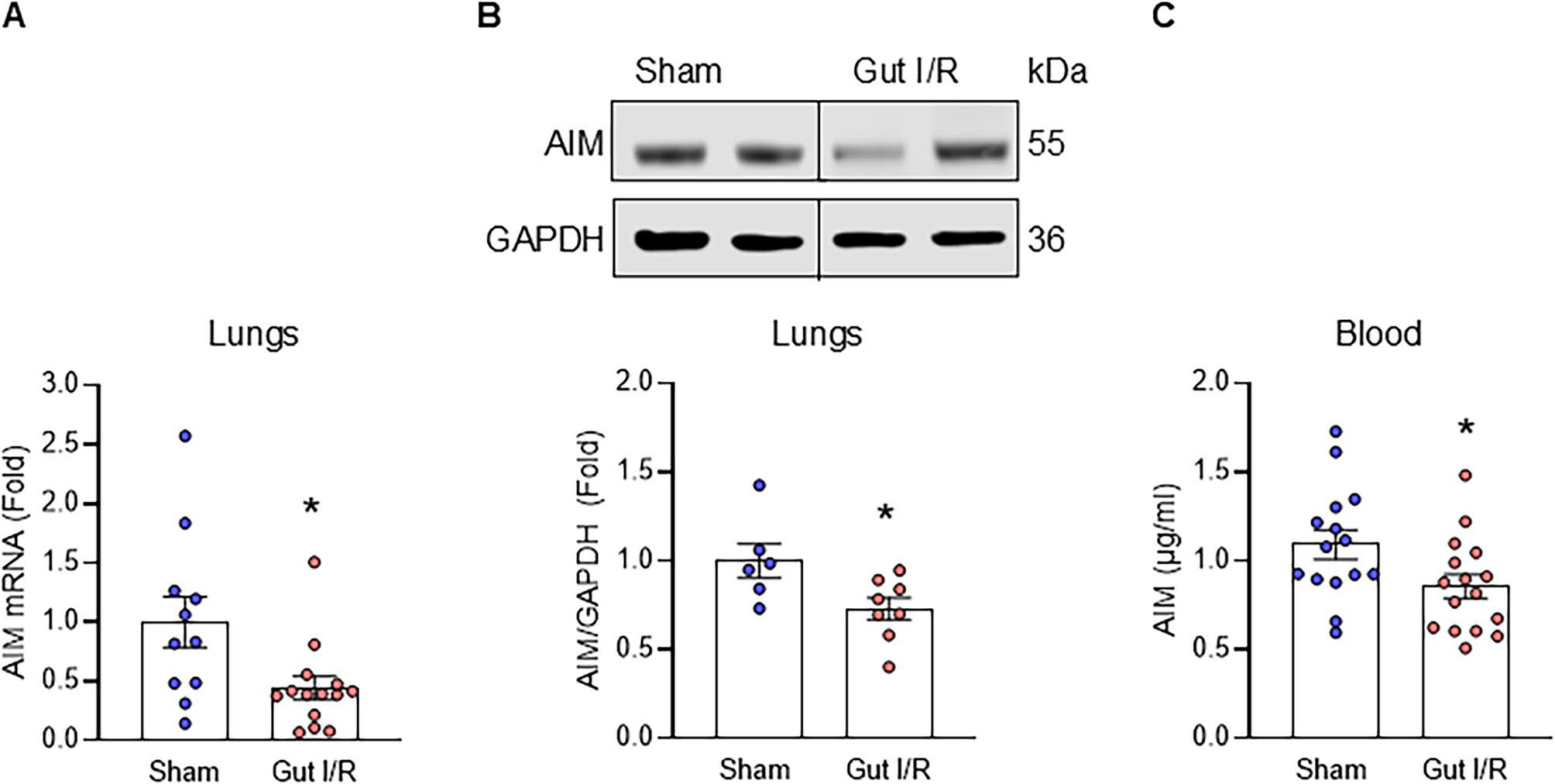
AIM is decreased after gut ischemia/reperfusion in male mice. Male mice underwent gut ischemia/reperfusion (I/R) through occlusion of the SMA for 60 min followed by 4 h of reperfusion before collecting lungs and plasma. (A) mRNA expression was evaluated in the lungs (*n* = 11–14/group, data combined from 4 independent experiments) by qPCR and is shown as fold expression over sham using β-actin as the reference gene. (B) Protein expression was determined by Western blotting for the lungs (*n* = 6–8/group, data combined from 3 independent experiments) and is reported as fold expression over sham using GAPDH as the reference gene. Representative blots are shown. **(C)** Plasma concentration of AIM were determined by ELISA (*n* = 15–16/group, data combined from 6 independent experiments). Data are expressed as mean ± SEM and compared by Student’s unpaired t-test (p*<0.05)

To determine the effect of gut I/R injury on the status of AIM, we assessed mRNA expression and protein levels in the lungs, which represent a critical area of organ injury. AIM mRNA expression markedly decreased by 55.9% in the lungs (*p* = 0.018) after gut I/R (Figs. 1A). In addition, protein levels decreased by 26.9% in the lungs (*p* = 0.032; Fig. 1B). After showing the impact of gut I/R on AIM in the lungs, we evaluated the concentration of AIM in the plasma given the systemic nature of reperfusion pathophysiology. Plasma concentrations of AIM were reduced by 22% in the mice exposed to gut I/R compared to sham (*p* = 0.0326; Fig. 1C). These data suggest that AIM is decreased after gut I/R in several areas of the disease, including the lungs and blood.

### AIM reduces eCIRP-induced cytokine expression in macrophages

**Fig. 2:**
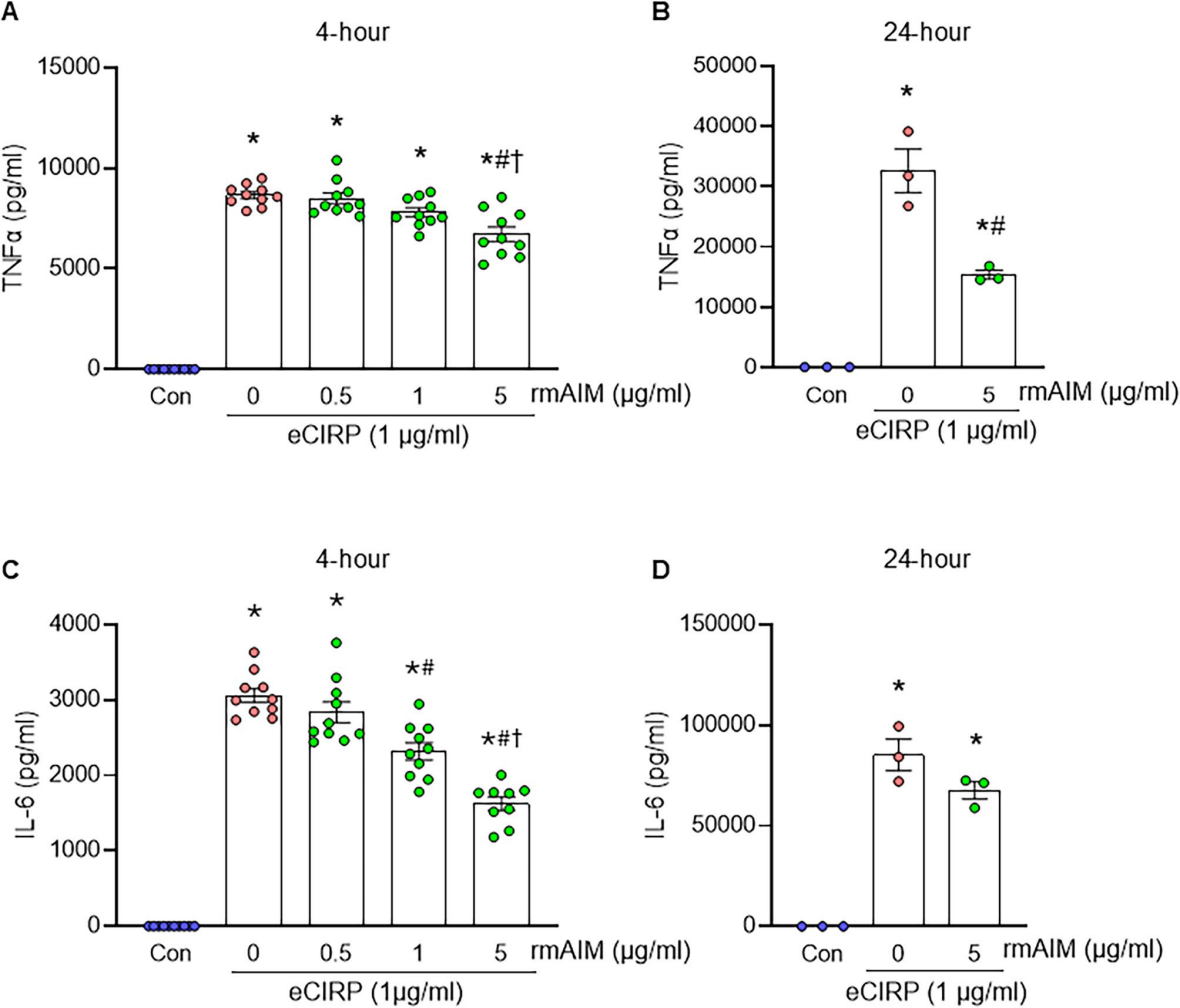
AIM treatment of macrophages reduces eCIRP-induced cytokine expression in vitro. Peritoneal cells from male mice were seeded at a concentration of 50,000/100 µl. Macrophages were isolated via the adhesion method and cultured overnight. eCIRP (1 µg/ml) and rmAIM were co-incubated for 30 min before treating macrophages for 4–24 h. **(A**,** B)** TNFα and **(C**,** D)** IL-6 expression were assessed via ELISA of the supernatant (*n* = 7–8/group for 4 h, *n* = 3/group for 24 h). Data are expressed as mean ± SEM and compared by one-way ANOVA and Tukey’s multiple comparison tests (*p < 0.05 vs. Control, ^#^*p* < 0.05 vs. 0 µg/ml rmAIM (eCIRP alone), ^†^*p* < 0.05 vs. 0.5 µg/ml rmAIM)

After observing reduced protein levels and mRNA expression of AIM in remote and systemic areas of gut I/R injury, we sought to evaluate the effects of rmAIM treatment. We evaluated AIM’s role in the inflammatory response of macrophages, which are key drivers of the innate immune response in gut I/R and known targets of AIM. Primary peritoneal cavity macrophages stimulated with eCIRP dramatically increased secretion of TNFα compared to control macrophages after 4 h (*p* < 0.0001) and 24 h (*p* < 0.0001). However, rmAIM treatment resulted in a dose-dependent reduction of TNFα (Fig. 2A, B). A dose of 5 µg/ml showed the most significant decrease of 22% at 4 h (*p* = 0.021), and 52% at 24 h (*p* = 0.0009). In addition to TNFα, IL-6 expression also decreased with rmAIM treatment (Fig. 2C, D). At both 4 and 24 h, eCIRP stimulation increased IL-6 concentrations significantly (*p* < 0.0001), which were then attenuated by rmAIM treatment. After 4 h, 5 µg/ml of rmAIM resulted in a 46.9% decrease in IL-6 (*p* < 0.0001) and at 24 h, a 20.6% decrease was observed (*p* = 0.119). While rmAIM had a more dramatic effect on TNFα after 24 h, IL-6 expression was affected at a much earlier timepoint. These results may be due to peak release times of the respective cytokines or may have implications on optimal dose frequency. At either time point, expression of both cytokines decreased, and with these results, rmAIM has demonstrated its ability to modulate the pro-inflammatory response of eCIRP-stimulated macrophages from male mice.

### AIM reduces eCIRP-induced cytokine expression in intestinal epithelial cells

**Fig. 3:**
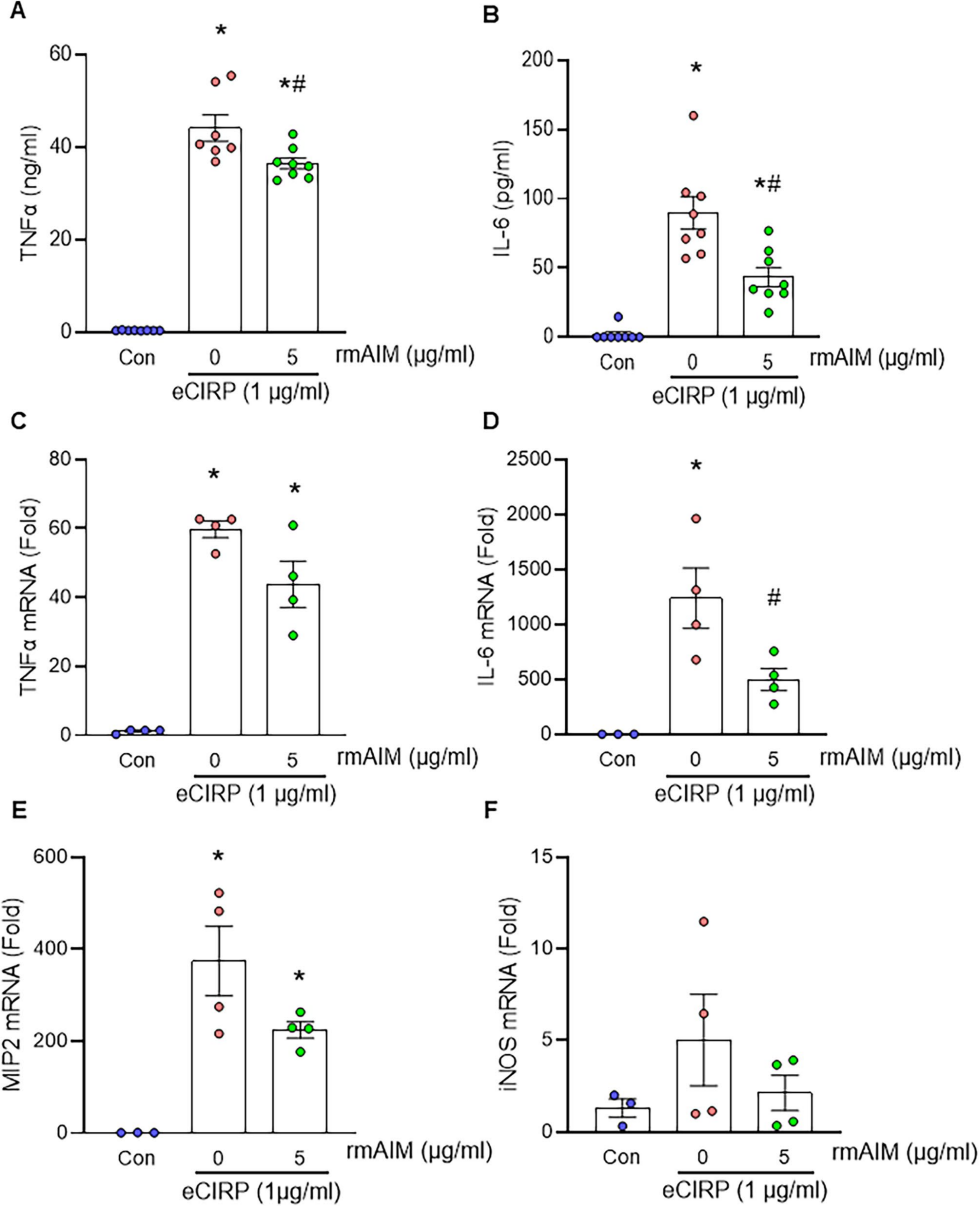
AIM treatment of intestinal epithelial cells reduces eCIRP-induced cytokine expression. IEC-6 cells were cultured at a concentration of 100,000/100 µl. eCIRP (1 µg/ml) and rmAIM (5 µg/ml) were co-incubated for 30 min before treatment for 24 h. **(A)** TNFα and **(B)** IL-6 expression were assessed via ELISA of the supernatant (*n* = 7–8/group). **(C-F)** mRNA expression was evaluated by qPCR (*n* = 4/group) of inflammatory markers **(C)** TNFα, **(D)** IL-6, **(E)** MIP2, and **(F)** iNOS. Data are expressed as mean ± SEM and compared by one-way ANOVA and Tukey’s multiple comparison tests (*p < 0.05 vs. Con, ^#^*p* < 0.05 vs. 0 µg/ml rmAIM (eCIRP alone)

Since rmAIM attenuated cytokine expression in eCIRP-stimulated macrophages, we then proceeded to evaluate rmAIM’s effects on another important cell type in gut I/R, intestinal epithelial cells. In these cells, a substantial increase in TNFα production was observed with eCIRP stimulation compared to control samples (Fig. 3A). However, TNFα decreased by 17.2% when IEC-6 cells were treated with rmAIM (*p* = 0.011). Along with TNFα, IL-6 demonstrated a similar anti-inflammatory response (Fig. 3B). Supernatant IL-6 concentration increased by 48-fold from the control to the eCIRP-stimulated group (*p* < 0.0001) with a subsequent decrease by 51.6% after rmAIM treatment. In addition to determining rmAIM’s influence on the production of pro-inflammatory cytokines, we also examined gene expression of important inflammatory mediators. In Fig. 3C-F, mRNA expression of TNFα, IL-6, MIP2, and iNOS are shown. Compared to the control group, mRNA expression in all four genes dramatically increased with eCIRP stimulation but then decreased with rmAIM treatment. Altogether, rmAIM treatment of intestinal epithelial cells results in the mitigation of pro-inflammatory cytokine expression and downregulation of mRNA for several genes associated with pro-inflammatory processes. Therefore, rmAIM can affect not only macrophages but also non-immune cells associated with gut I/R pathology.

### AIM recovers macrophage metabolism that is disrupted by eCIRP stimulation

**Fig. 4:**
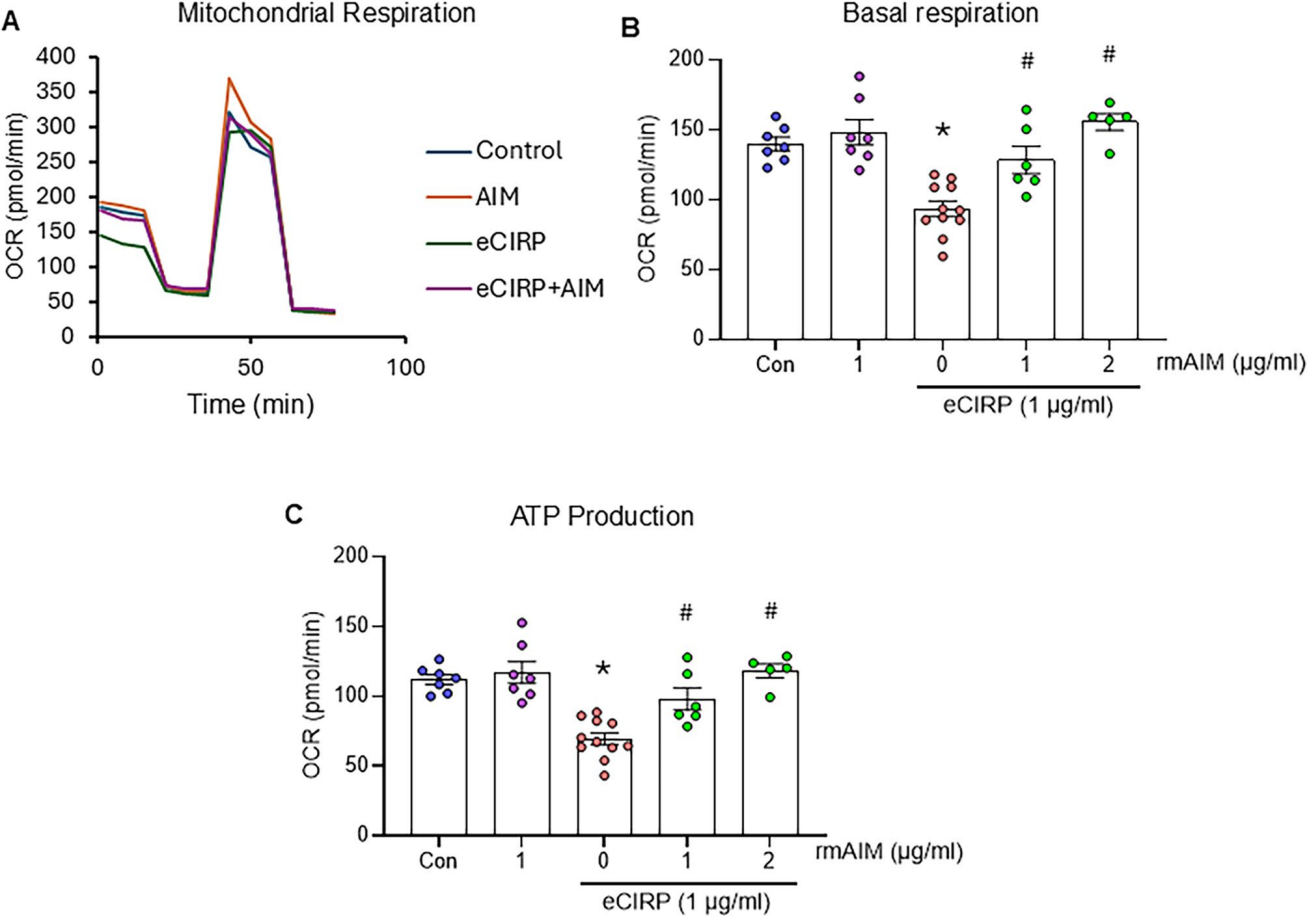
AIM recovers macrophage metabolism that is disrupted by eCIRP stimulation. RAW 264.7 cells (20,000/100 µl) were treated with eCIRP (1 µg/ml) and/or rmAIM in various doses for the Seahorse XF Mito Stress Test assay. **(A-C)** Metabolic activity was assessed by oxygen consumption rate (OCR) with the Seahorse XF Pro Analyzer and Seahorse Wave Pro Software (*n* = 5–11/group): **(A)** test profile, **(B)** basal respiration, **(C)** ATP production. OCR (pmol/min) was compared across all groups, and data are expressed as mean ± SEM and compared by one-way ANOVA and Tukey’s multiple comparison tests (*p < 0.05 vs. Control, ^#^*p* < 0.05 vs. 0 µg/ml rmAIM (eCIRP))

After demonstrating a reduction in cytokine expression with treatment of rmAIM in both macrophages and intestinal epithelial cells, we evaluated the metabolic function of macrophages stimulated by eCIRP with or without rmAIM using the Seahorse XF Mito Stress Test assay. Mitochondrial respiration in macrophages provides information on the overall functional health of these immune cells and can indicate whether they have pro or anti-inflammatory phenotypes (Pérez and Rius-Pérez 2022). RAW 264.7 macrophages treated with eCIRP had impaired mitochondrial function compared to PBS-treated macrophages, and mitochondrial function was recovered with treatment of rmAIM in a dose-dependent fashion. Figure 4A shows the overall pattern of mitochondrial function, represented by oxygen consumption rate (OCR), under basal levels and under stress. Figure 4B, C quantifies mitochondrial activity by OCR for each group in basal respiration, maximal respiration, and ATP production, respectively. Compared to control, macrophages treated with eCIRP alone had a significantly lower OCR at basal levels (Fig. 4B). However, macrophages treated with 2 µg/ml rmAIM in addition to eCIRP had higher rates of oxygen consumption by 66.7% vs. eCIRP alone, demonstrating improved mitochondrial function (*p* < 0.0001). In Fig. 4C, macrophages treated with eCIRP alone decreased ATP production by 38% (*p* < 0.0001), which was increased by 70.2% with rmAIM (*p* < 0.0001). These data indicate that eCIRP negatively affects mitochondrial function, whereas AIM can counteract eCIRP’s effects to recover mitochondrial activity.

### AIM interacts with eCIRP receptors in silico

**Fig. 5:**
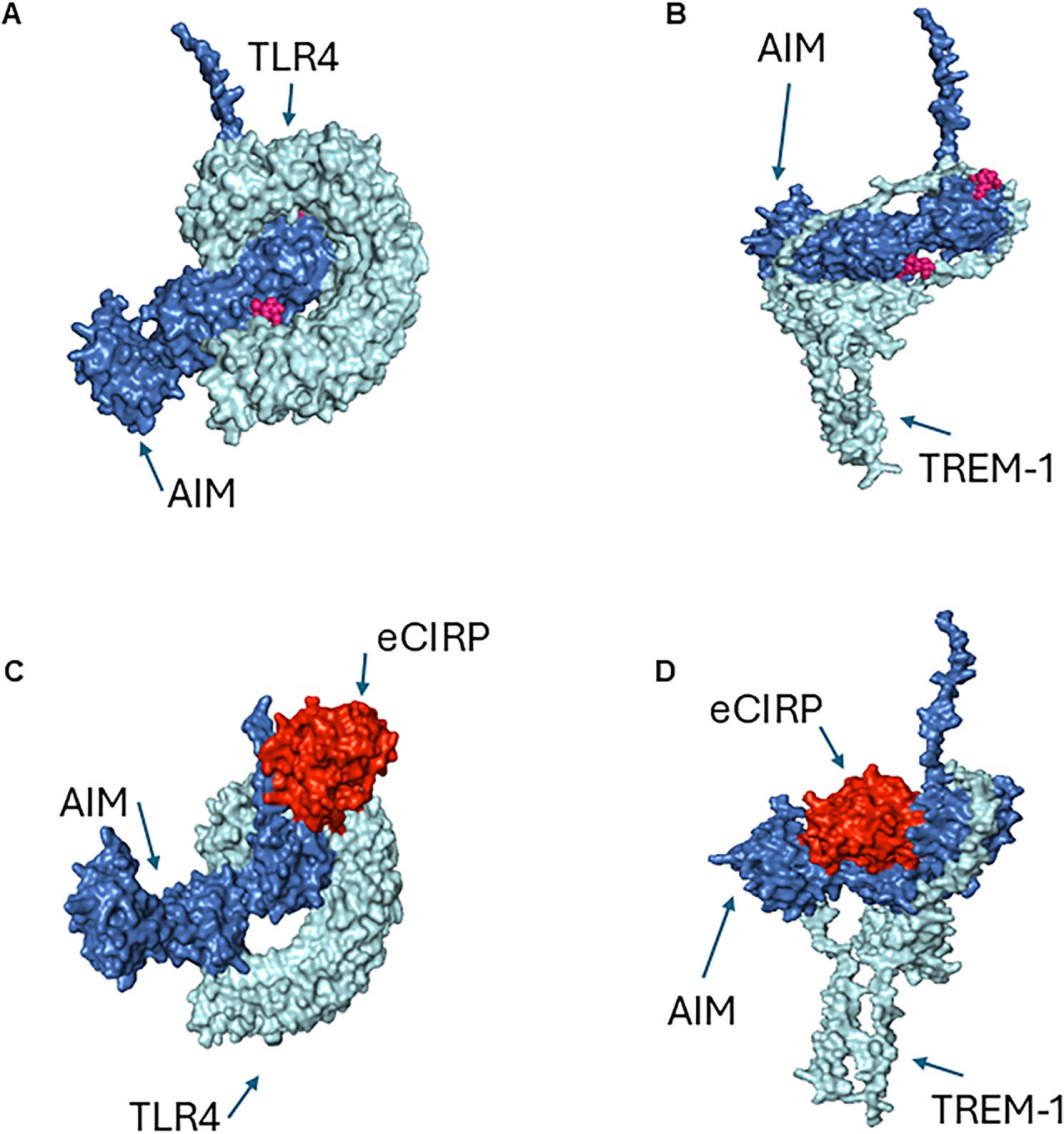
AIM shows interaction with eCIRP receptors *in silico.* Theoretical interactions among AIM, eCIRP’s target receptors (TLR4 and TREM-1), and eCIRP were evaluated in silico by computational modelling using the Uniprot database. Models of AIM interacting with **(A)** TREM-1, **(B)** TLR4, **(C)** the eCIRP-TLR4 complex, and **(D)** the eCIRP-TREM-1 complex are shown

After demonstrating that AIM decreases after gut I/R and that treatment with rmAIM counteracts eCIRP’s pro-inflammatory effects, we then evaluated AIM’s interactions with eCIRP and eCIRP’s receptors. We performed in silico modelling to determine potential mechanisms through which AIM inhibits eCIRP’s effects (Fig. 5A-D). The interaction between AIM and eCIRP was predicted to be nonspontaneous and unfavorable with a positive binding energy and negative free energy of dissociation, implying that this interaction was unlikely to be the mechanism (Table 1). However, AIM has the potential to bind TLR4 and TREM1 to interrupt eCIRP activation of these receptors (Figure A, B). Both interactions were predicted to be spontaneous with negative binding energies and stable with positive free energies of dissociation (Table 2**)**. Compared to TREM-1-AIM, the TLR4-AIM interaction was predicted to have a lower binding energy and higher free energy of dissociation for more spontaneous and stable binding (Table 2). The complex of eCIRP binding to AIM and TLR4 is predicted to be spontaneous but with a higher binding energy and lower free energy of dissociation than AIM and TLR4 without eCIRP (Fig. 5C; Table 2). eCIRP binding with AIM and TREM-1 is predicted to have a low free energy of dissociation, indicating a more transient, less favorable interaction than TREM-1 and AIM without eCIRP (Fig. 5D; Table 2). In conclusion, our modelling predicts that AIM attenuates eCIRP-induced inflammation through binding target receptors. This binding likely dampens activation of downstream pathways and thus reduces cytokine production and skews macrophages away from an inflammatory phenotype.

**Table 1:** AIM is predicted to have an unfavorable interaction with eCIRP. Computational modeling of AIM docked on eCIRP with predicted interaction metrics

Complex	Surface Area (Ȧ^2^)	Binding Energy (^∆i^G) Kcal/mol	Free Energy of Dissociation (∆G^diss^) Kcal/mol	Entropy Change at Dissociation (T∆S^diss^)	*N*_HB_	*N*_SB_
AIM-eCIRP	690.2	3.8	−14.8	12.8	4	0

**Table 2:** AIM is predicted to have favorable interactions with eCIRP receptors, but complexes with eCIRP are less favorable. Computational modeling of AIM docked on TLR4 and TREM-1 receptors as well as eCIRP docked on the AIM-TLR4 and AIM-TREM-1 complexes with predicted interaction metrics

Complex	Surface Area (Ȧ^2^)	Binding Energy (^∆i^G) Kcal/mol	Free Energy of Dissociation (∆G^diss^) Kcal/mol	Entropy Change at Dissociation (T∆S^diss^)	*N*_HB_	*N*_SB_
AIM-TLR4	2272.2	−29.9	13.9	16.4	0	0
AIM-TREM-1	1663.0	−21.0	5.9	15.4	0	0
eCIRP-AIM-TLR4	7684.0	−9.8	7.5	15.4	21	9
eCIRP-AIM-TREM-1	7793.1	−6.1	−19.6	27.4	0	0

**Fig. 6:**
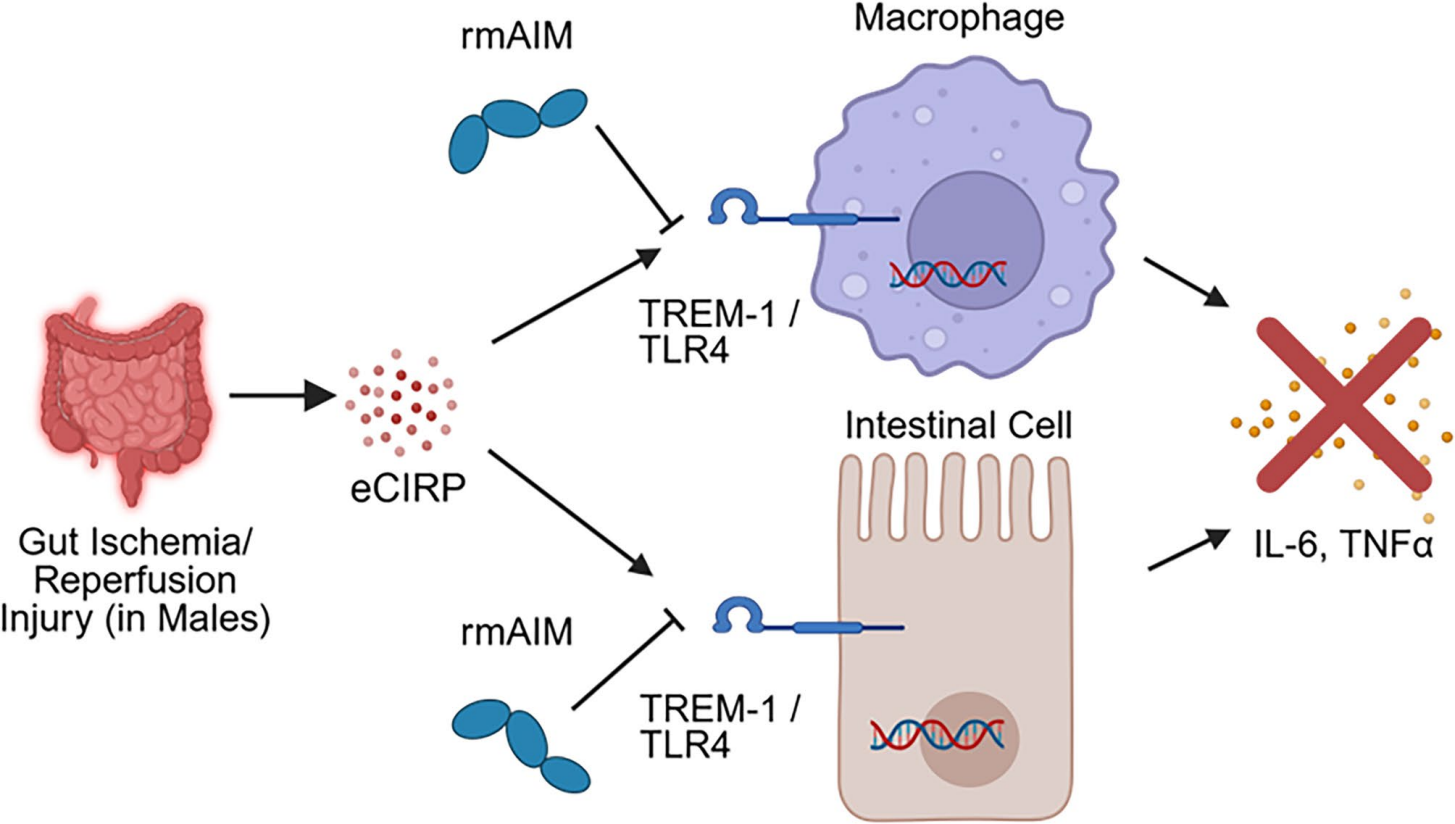
Findings schema. Gut ischemia/reperfusion (I/R) injury releases eCIRP systemically in male mice. Recombinant AIM (rmAIM) reduces cytokine expression and inflammation by binding to TLR4 and TREM-1, thereby inhibiting ligand-receptor interactions in macrophages and intestinal epithelial cells and reducing the expression of pro-inflammatory cytokines

## Discussion

In this study, we have shown that AIM protein levels and mRNA expression are markedly decreased in the blood and lungs of mice after gut I/R. We then explored the effects of rmAIM on macrophages (resident immune cells of the lamina propria of the intestine) and epithelial cells, which maintain barrier function in homeostasis. Both populations play a vital role in gut I/R pathophysiology. Treatment of eCIRP-stimulated macrophages and intestinal epithelial cells with rmAIM leads to decreased expression of pro-inflammatory cytokines. These findings indicate a reduction in M1 polarization. Macrophages also demonstrate recovered metabolic activity with rmAIM treatment. The mechanism of metabolic recovery is likely through reduction of eCIRP-induced mitochondrial DNA fragmentation, which occurs via TLR4/MyD88 signaling (Li et al. 2017). Lastly, we used computational modelling to investigate a potential mechanism for rmAIM as an inhibitor of eCIRP activity. These findings demonstrate the possibility of AIM as a novel therapy in gut reperfusion injury in males (Fig. 6), a condition that lacks effective treatment despite decades of understanding the disease.

Acute gut ischemia is a devastating pathology that requires prompt recognition and treatment (Clair and Beach 2016; Ehlert 2018). Even with expedient resuscitation, embolectomy or thrombectomy, and surgical exploration, reperfusion injury can negatively impact the patient’s recovery and prognosis (Cowled and Fitridge 2011). Reperfusion injury is a complex phenomenon that releases several byproducts of cellular stress, including reactive oxygen species, electrolytes, and cytokines (Cowled and Fitridge 2011; Zhang et al. 2024). However, eCIRP, has been shown to significantly influence the severity of the disease, making neutralization of eCIRP a viable strategy (Cen et al. 2017).

eCIRP was originally discovered as a DAMP in critically ill patients and in mouse models of sepsis and hemorrhagic shock (Qiang et al. 2013). eCIRP first demonstrated binding affinity to TLR4, activating NF-κB to promote inflammation (Qiang et al. 2013). Further studies implicated eCIRP in various other pathologies, such as I/R injury, and as a ligand for TREM-1 (Denning et al. 2020a; Denning et al. 2020 b). While other DAMPs have been evaluated as targets of AIM, eCIRP has not previously been explored (Oliveira et al. 2024). The ability of AIM to reduce cytokine concentration in the supernatant of eCIRP-stimulated cells indicates its potential as a novel anti-eCIRP strategy. Mechanistically, in silico evaluation of AIM predicts an ability for the glycoprotein to interrupt eCIRP’s interaction with key receptors.

AIM is a glycoprotein primarily synthesized and secreted by macrophages, including peritoneal macrophages and those found in lymphatic tissues (Oliveira et al. 2024; Sanjurjo et al. 2015). Additionally, there is some evidence that epithelial cells, such as alveolar type II cells, secrete AIM (Sanjurjo et al. 2015). The glycoprotein exercises a variety of immunologic and metabolic functions in numerous organ systems (Sanchez-Moral et al. 2021). Published data on the half-life of AIM is sparse. However, there is literature showing a sharp increase in AIM protein levels 6 h after rmAIM treatment with a return to baseline 24 h later in both the serum and peritoneal cavities of mice (Oliveira et al. 2024). Furthermore, improvement in survival in a CLP model of sepsis was observed up to 9 days beyond the initial injection, suggesting a favorable biological half-life for use as a therapeutic (Oliveira et al. 2024).

In a previous study, treatment with AIM in sepsis showed a negative correlation with protein levels of HMGB1, another prominent DAMP (Oliveira et al. 2024). Additionally, AIM can bind to DAMPs, such as S100 proteins, heat shock proteins, and HMGB1, to reduce inflammation and improve outcomes in mouse models of stroke (Maehara et al. 2021). While the presence of AIM was correlated with decreased binding to Receptor for Advanced Glycation Endproducts (RAGE) by HMGB1, the affinity of HMGB1 to AIM was relatively low (Maehara et al. 2021). It is possible that AIM binds equally or even more potently to HMGB1’s receptor than the DAMP itself. However, AIM’s interaction with RAGE or other receptors was not explored. In our evaluation, we found that AIM was not predicted to bind to eCIRP spontaneously in silico but rather to eCIRP’s target receptors, TLR4 and TREM-1. In either mechanism, there is solid evidence of AIM’s DAMP-neutralizing capabilities. Beyond eCIRP, a previous study also showed that AIM can reduce cytokine production from other TLR4 agonists, such as LPS and Pam3 (Sanjurjo et al. 2015). While their focus was on the role of CD36, the authors note that given CD36’s activity in TLR signaling, it is likely that AIM interferes with TLR ligand recognition, as we propose in our study.

The benefits of AIM in acute inflammation are not without controversy, however. Studies evaluating serum AIM concentration in patients with sepsis and systemic lupus erythematosus (SLE) found increases in AIM concentration compared to healthy subjects (Gao et al. 2019a; Lai et al. 2018). However, studies of mouse models, which evaluate different tissues beyond serum, showed decreases in tissue and serum AIM early after the onset of sepsis (Oliveira et al. 2024). AIM protein levels did increase in the peritoneum, though, indicating a possible redistribution of this anti-inflammatory protein rather than strict decrease or increase (Oliveira et al. 2024). AIM protein levels may also differ depending on the time of collection and the underlying pathology. In our study, tissue protein levels and mRNA expression in the lungs as well as plasma concentrationwere reduced. AIM likely decreases in gut I/R both due to consumption of endogenous supplies of AIM through the innate immune response and the dysfunction or death of AIM-producing cells, namely macrophages and epithelial cells of the lungs. Therefore, administration of recombinant forms of AIM are needed to promote its anti-inflammatory activities.

While many studies report the beneficial effects of AIM in several disease models (Arai et al. 2016; Lee et al. 2021; Li et al. 2020; Maehara et al. 2021; Oliveira et al. 2024), other studies report detrimental effects (Castelblanco et al. 2021; Gao et al. 2019b). For example, AIM clears fungi in peritonitis and removes cell debris in acute kidney injury (Arai et al. 2016; Tomita et al. 2017). AIM also downregulates inflammation through autophagy in acute and chronic liver injury models (Li et al. 2020; Sanchez-Moral et al. 2021). In other scenarios, authors have reported detrimental effects of AIM. In cardiovascular disease, AIM can perpetuate the lifespan of plaque-promoting foam cells, and in pneumonia, AIM can reduce intracellular killing of *S. aureus* in pneumonia (Gao et al. 2019b; Sanchez-Moral et al. 2021). In pneumonia, the authors hypothesized that AIM may allow bacteria to persist due to the glycoprotein’s antiapoptotic functions (Gao et al. 2019). Our study focuses on sterile inflammation, which would not involve bacteria. Furthermore, in our experiments, AIM’s anti-apoptotic functions do not appear to be a primary factor in the observed reduction of inflammation, as demonstrated by similar gene expression of ZBP1, a PANoptosis marker, in both eCIRP and rmAIM groups (data not shown). These differences may explain AIM’s consistent reduction of cytokines across cell types in our eCIRP stimulation models and highlights the importance of evaluating AIM’s function in specific disease models to determine harm or benefit.

Although the data in this study demonstrate reduced protein levels and mRNA expression of AIM in acute gut I/R and the potential for AIM as an anti-eCIRP therapy, these findings are not without limitations. For example, while the protein levels of AIM in the lungs and blood are significantly decreased, we did not observe the same decrease in the intestine. The effects of gut ischemia on the intestine can often be heterogeneous based on characteristics of the different segments and collateral blood supply, resulting in a patchy necrosis before the uniformly lethal necrosis of the entire intestine. This aspect of the intestinal response to gut ischemia compared to the more evenly distributed lung injury after reperfusion may explain the higher variability in the intestinal AIM protein levels compared to lung AIM protein levels after gut I/R. In addition, AIM concentrations have considerable variability in human studies as well (Oliveira et al. 2024). Furthermore, this study only evaluated male mice due to the differences in immune response between males and females (Homma et al. 2005; Hundscheid et al. 2020; Wu et al. 2021). Therefore, the presented findings only apply to male subjects. Also, while the effects of AIM on macrophages and intestinal epithelial cells were evaluated, the effects of AIM on vascular endothelial cells, which also play a key role in I/R injury, were not assessed. AIM’s impact on endothelial cells would likely follow a similar trend. Future studies should focus on the role of AIM in females as well as its effects on vascular endothelial cells. Finally, this study does not provide in vivo therapeutic data, which limits our ability to determine its efficacy in gut I/R. However, the decreases in AIM plasma concentration, protein levels in the lung, and mRNA expression observed during gut I/R as well as the anti-inflammatory effects observed in vitro highlight AIM’s importance in the innate immune response to sterile inflammation.

## Conclusion

In conclusion, this study is the first to evaluate AIM protein levels, mRNA expression, and plasma concentration in males with gut I/R and the first to evaluate AIM’s effects on eCIRP-induced inflammation. Therefore, this study lays important groundwork for the continued evaluation of AIM as an eCIRP-neutralizing therapy, which broadens its applications to all pathologies that are exacerbated by eCIRP. Additional studies investigating AIM in patients after gut ischemia, rmAIM treatment of male and female mice after gut I/R, and AIM’s interaction with TLR4 and TREM-1 are needed.

## Supplementary Information

Supplementary Material 1.

## Data Availability

No datasets were generated or analysed during the current study.

## Abbreviations

AIM: Apoptosis inhibitor of macrophage
ARDS: Acute respiratory distress syndrome
DAMP: Damage associated molecular pattern
DMEM: Dulbecco’s Modified Eagle Medium
eCIRP: Extracellular cold inducible RNA-binding protein
FBS: Fetal bovine serum
H: Hour
I/R: Ischemia/reperfusion
I-TASSER: Iterative Threading Assembly Refinement
KIM-1: Kidney injury molecule 1
Min: Minute
OCR: Oxygen consumption rate
PBS: Phosphate-Buffered saline
PCN/Strep: Penicillin/streptomycin
RAGE: Receptor for advanced glycation end products
RIPA: Radioimmunoprecipitation assay
Rm: Recombinant murine
RPMI: Roswell Park Memorial Institute
SLE: Systemic lupus erythematosus
SMA: Superior mesenteric artery
SRCR: Scavenger receptor cysteine-rich
TLR4: Toll-like receptor 4
TREM-1: Triggering receptor expressed on myeloid cells-1
