# Supplementary figures and images for: The role of an anti-inflammatory molecule AIM/CD5L in gut ischemia/reperfusion injury of male mice
Source: Mol Med. 2025 Oct 29;31:321. doi: 10.1186/s10020-025-01385-1 (PMC12574078; doi:10.1186/s10020-025-01385-1)

## Slide 1
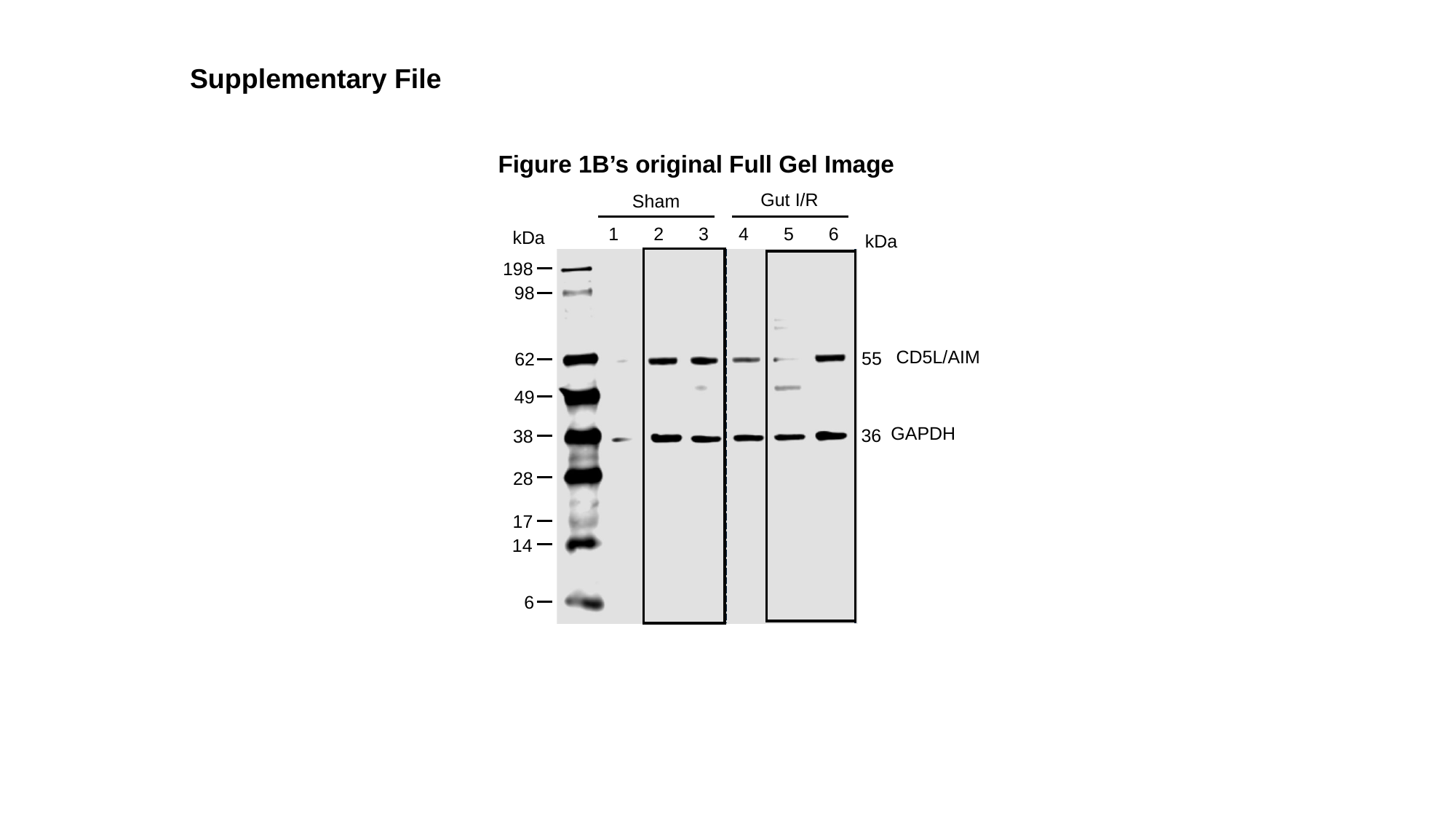

Supplementary File
Figure 1B’s original Full Gel Image
Gut I/R
Sham
1
2
3
4
5
6
kDa
kDa
198
98
CD5L/AIM
55
62
49
GAPDH
36
38
28
17
14
6

Supplement: Supplementary file 1 — Supplementary Material 1. [file 10020_2025_1385_MOESM1_ESM.pptx]
